# Spin polarized density functional theory calculations of the electronic structure and magnetism of the 112 type iron pnictide compound $\hbox {EuFeAs}_2$

**DOI:** 10.1038/s41598-021-91301-4

**Published:** 2021-06-08

**Authors:** Farshad Nejadsattari, Zbigniew M. Stadnik

**Affiliations:** Department of Physics, University of Ottawa, Ottawa, ON K1N 6N5 Canada

**Keywords:** Physics, Condensed-matter physics, Electronic properties and materials

## Abstract

Using density-functional theory, we investigate the electronic, magnetic, and hyperfine-interaction properties of the 112-type iron-pnictide compound ${\hbox {EuFeAs}}_2$, which is isostructural to the high-temperature iron-based superconductor ${\hbox {Ca}}_{1-x}{\hbox {La}}_x{\hbox {FeAs}}_2$. We show that the band structure of ${\hbox {EuFeAs}}_2$ is similar to that of the 112-type compounds’ family, with hole-like and electron-like bands at the Brillouin-zone center and corners, respectively. We demonstrate that the bands near the Fermi level originate mainly from the Fe atoms. The presence of a mixture of ionic and covalent bonding is predicted from the charge-density and atom-resolved density-of-states calculations. There is good agreement between the calculated hyperfine-interaction parameters with those obtained from the $^{57}$Fe and $^{151}$Eu Mössbauer measurements. The spatial distribution of atoms in ${\hbox {EuFeAs}}_2$ leads to an in-plane 2D magnetism. Moreover, *ab*-*initio* calculations predict the compound’s magnetic moment and the magnetic moments of each constituent atom. Also, the density of states profile provides insight into the relative magnitude of these moments. Electronic structure calculations and Fermi surface topology reveal various physical and chemical properties of ${\hbox {EuFeAs}}_2$. Valence electron density maps indicate the co-existence of a wide range of chemical bonds in this system, and based on structural properties, the transport characteristics are deduced and discussed. A thorough analysis of the atomic structure of ${\hbox {EuFeAs}}_2$ and its role in the bond formation is presented.

## Introduction

Recently, the iron-based pnictides containing rare earth (RE) elements have been successfully synthesized, and their electronic and magnetic properties have been extensively studied. Among them, compounds belonging to the family of the 112-type iron-pnictide superconductors (Ca,RE)${\hbox {FeAs}}_2$ (RE = La, Pr, Nd, Sm, Gd) have been widely investigated^1–5^. In particular, density-functional theory calculations on these class of iron-based pnictides were performed. They suggest the existence of four hole-like and two electron-like bands intersecting the Fermi level ($E_{{\text {F}}}$) around, respectively, the $\Gamma$ and M points, which are related to the Fe 3*d* and As 4*p* orbitals^1^.

Very recently, a new Eu-containing iron-pnictide compound, ${\hbox {EuFeAs}}_2$, has been synthesized^6^. Its crystal structure is composed of two Eu planes, ${\hbox {Fe}}_2{\hbox {As}}_2$ layers, and zigzag chain layers of As^2,7^. There are two space groups in which ${\hbox {EuFeAs}}_2$ is reported to crystallize. The orthorhombic space group *Imm*2 was used in Ref. ^7^, and the monoclinic space group $P2_1$/*m* was employed in Refs. ^6,8^.

The presence of Eu and Fe in the ${\hbox {EuFeAs}}_2$ compound points potentially towards the existence of rich chemical and physical characteristics. These characteristics can be deduced from the electronic structure and fermiology of this compound. Also, ${\hbox {EuFeAs}}_2$ is expected to order magnetically. Indeed, its complex magnetism was studied recently experimentally^9^. However, there has been no first-principles theoretical study of the compound’s electronic structure and magnetism.

This work’s main objective is to study the origin of some of the physical properties of ${\hbox {EuFeAs}}_2$ via a detailed investigation of its electronic structure using the *ab*-*initio* density-functional theory calculations. The formation and the type of chemical bonds and the charge transport properties in this compound are investigated. The chemical bonds between the various atoms in ${\hbox {EuFeAs}}_2$ are found to be similar to those observed in similar compounds^1,10–14^. A thorough discussion of the Fermi surface topology allows for a better understanding of the electronic characteristics of ${\hbox {EuFeAs}}_2$. A comparison is made between the calculated physical quantities and those obtained from the magnetic and Mössbauer measurements. Moreover, with the aid of *ab*-*initio* calculations, one can compare the experimental results with those derived from computations.

## Theoretical methods

We carried out first-principles calculations of the electronic structure and Mössbauer hyperfine-interaction parameters of ${\hbox {EuFeAs}}_2$ in the context of density-functional theory employing the full-potential linearized augmented-plane-wave method that is implemented in the WIEN2k package^15^. This method is described in detail in Ref. ^16^. In this paper, the interstitial region’s valence wave functions are expanded in spherical harmonics up to $l = 4$. They are expanded to a maximum of $l = 12$ in the muffin-tin (MT) region. The MT radii used for Eu, Fe, and As were 2.50, 2.32, and 2.21 a.u., respectively. We used the generalized gradient approximation (GGA) scheme of Perdew et al.^17^ for the exchange-correlation potential. For the Eu 4*f* and Fe 3*d* states, the values of the effective Hubbard-like interaction energies of, respectively, 0.55 and 0.20 Ry, were used. For correlated systems and for a full potential calculation, we have used an *effective* *Hubbard* *parameter* defined as $U_{\text {eff}} = U - J$. It can be calculated with the augmented plane-wave methods. For the Fe 3*d* states, $U_{\text {eff}} = E_{3d\uparrow }(\frac{n+1}{2},\frac{n}{2})- E_{3d\uparrow }(\frac{n+1}{2},\frac{n}{2}-1)-E_{\text {F}}(\frac{n+1}{2},\frac{n}{2})-E_{\text {F}}(\frac{n+1}{2},\frac{n}{2}-1)$, where *n* is the orbital occupation number^18^. A similar relation holds for the Eu 4*f* states. The Hubbard potential of 0.27 Ry with a *J* constant of 0.065 Ry leads to an effective Hubbard parameter of 0.20 Ry for the Fe 3*d* states. A similar analysis for the Eu 4*f* states leads to the value of 0.038 Ry for the calculated *J* constant. Combined with the value of 0.59 Ry for the Hubbard parameter, it leads to the effective Hubbard potential of 0.549 Ry (7.48 eV). The plane-wave cut-off parameter was set to $R_{\text {MT}} \times K_{\text {MAX}} = 7.5$. Here, $R_{\text {MT}}$ is the smallest MT radius in the unit cell and $K_{\text {MAX}}$ is the maximum *K* vector used in the plane-wave expansion in the interstitial region. One thousand three hundred thirty-one inequivalent *k*-points were used within a $21 \times 21\times 21$ *k*-mesh in the irreducible wedge of the first Brillouin zone. The calculations were done for the experimental lattice and atomic position parameters^7^.

## Results and discussion

### Crystal structure

The most probable space group in which the ${\hbox {EuFeAs}}_2$ compound crystallizes is *Imm*2 (No. 44)^7^. The relevant crystal structure parameters of ${\hbox {EuFeAs}}_2$ are given in Table 1.

**Figure 1:**
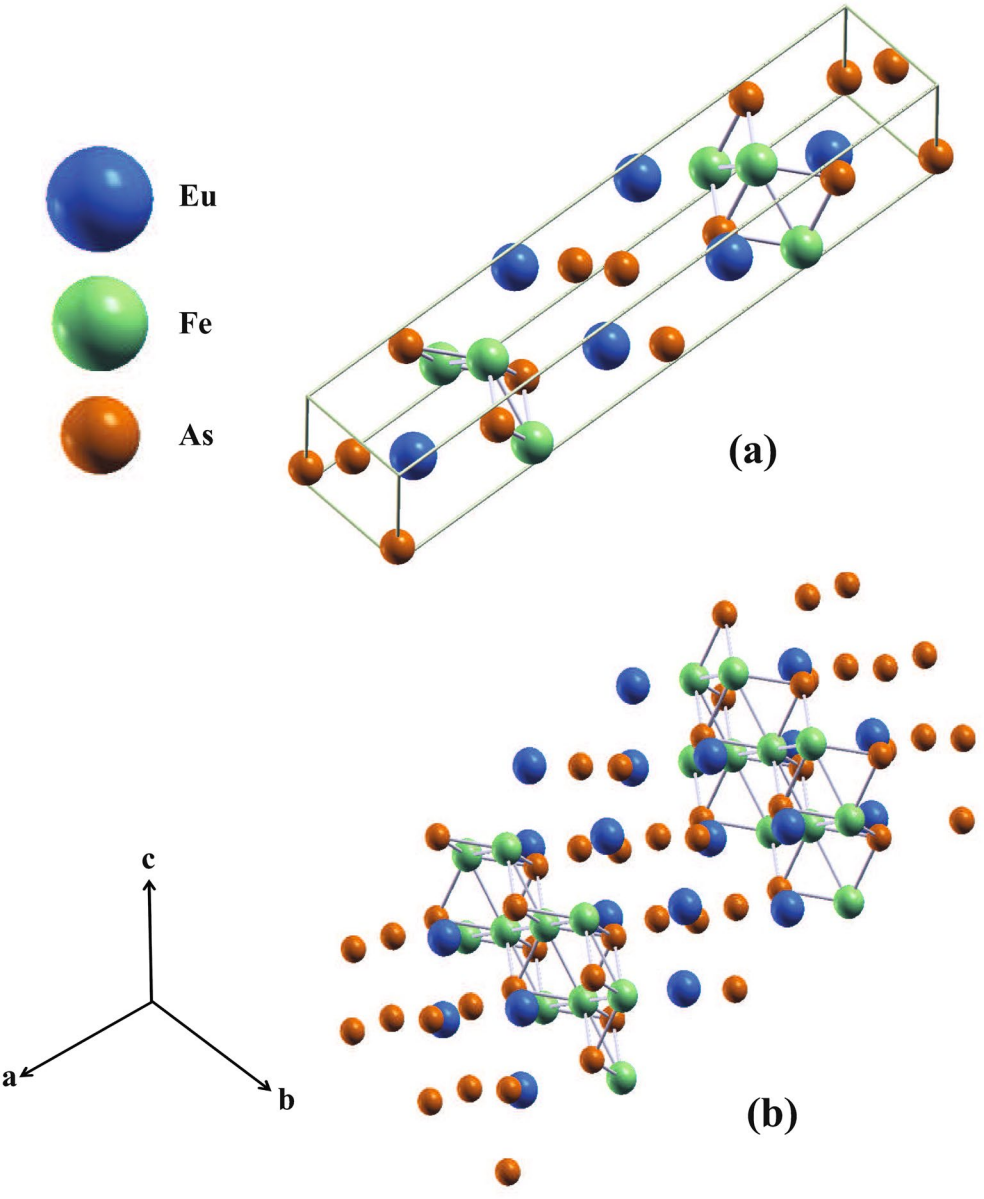
The unit cell (**a**) and the layered structure (**b**) of ${\hbox {EuFeAs}}_2$. SigmaPlot v14, www.systatsoftware.com.

Figure 1 shows the crystal structure of ${\hbox {EuFeAs}}_2$. The interactions between the constituent atoms are shown by the rods connecting these atoms. Each unit cell (Fig. 1a) contains four formula units of ${\hbox {EuFeAs}}_2$. The unit cell’s dimensions and the atoms’ spatial distribution play an important role in the studied compound’s electronic properties. The unit cell of ${\hbox {EuFeAs}}_2$ is in the form of a cuboid (Fig. 1a), which is highly elongated along the *a* direction ($a\gg b,c$, Table  1). The ${\hbox {FeAs}}_2$ units in each unit cell are greatly separated along the *a* direction, and, consequently, the interactions are small compared to those along other directions. Moreover, no magnetic coupling is expected to exist along the *a* direction. Consequently, any possible magnetic order in this compound will be two-dimensional and restricted to the *bc* plane. The $a\gg b,c$ relationship also indicates that the two-dimensional characteristics in ${\hbox {EuFeAs}}_2$ will be more prominent than those in other Fe-based pnictides.

The Fe and ${\hbox {As}}_1$ atoms in the unit cell (Fig. 1a) are shown to be connected by thin rods, which represents the covalent bonds within the ${\hbox {FeAs}}_2$ units. Covalent bonds within these units are favoured because of the close distance between the Fe and ${\hbox {As}}_1$ atoms. The ${\hbox {FeAs}}_2$ units are separated by two sheets of the Eu atoms and sheets of the ${\hbox {As}}_{2,3}$ atoms, which act as insulating layers.

Figure 1b displays multiple unit cells further to indicate the blocks of the ${\hbox {FeAs}}_2$ units. The insulating sheets of the Eu and ${\hbox {As}}_{2,3}$ atoms separating these units are depicted in this figure. One can consider the Eu and ${\hbox {As}}_{2,3}$ sheets as barriers for the conduction electrons forbidding them to propagate freely along the *a* direction. The absence of connecting rods between the Eu and ${\hbox {As}}_{2,3}$ atoms indicates the existence of ionic bonding between them. Consequently, the dominant insulating behaviour of ${\hbox {EuFeAs}}_2$ along the *a* direction results from the formation of ionic bonds to a greter extent and covalent bonds to a lesser extent between the Eu counterions and the Fe-As system.

Based on electron density calculations (*vide infra*), valence charge delocalization is also present between the ${\hbox {As}}_2$ and ${\hbox {As}}_3$ atoms. Moreover, as the Eu atoms in ${\hbox {EuFeAs}}_2$ exist in the divalent state^9^, they transfer their 6*s* electrons to the ${\hbox {As}}_{2,3}$ atoms and the Fe–${\hbox {As}}_1$ units, thereby forming strong ionic bonds. The Fe–${\hbox {As}}_1$ bond length and the bond angle ${\hbox {As}}_1$–Fe–${\hbox {As}}_1$ in ${\hbox {EuFeAs}}_2$ are, respectively, 2.4122 Å and $108.212^\circ$. It has been suggested that the bond angle correlates with the transition temperature, $T_{\text c}$, in Fe-based superconductors^19^. The highest $T_{\text c}$ seems to be achieved in the structures with the tetrahedron angle closest to the ideal tetrahedron angle of $109.47^\circ$. This suggestion may be consistent with the value of $T_{{\text c}} = 13.8$ K in the ${\hbox {EuFe}}_{0.97}{\hbox {Ni}}_{0.03}{\hbox {As}}_2$ superconductor^8,9^.

There is a strong structural similarity between ${\hbox {EuFeAs}}_2$ and ${\hbox {Ca}}_{1-x}{\hbox {La}}_x{\hbox {FeAs}}_2$. The ${\hbox {As}}_2$ and ${\hbox {As}}_3$ atoms form zigzag chains, and their distances are classified into the short and the long ones. In ${\hbox {EuFeAs}}_2$, the short ${\hbox {As}}_2$-${\hbox {As}}_3$ distance is 2.5568 Å and the corresponding ${\hbox {As}}_3$-${\hbox {As}}_2$-${\hbox {As}}_3$ angle is $99.688^\circ$, with the short-distance ${\hbox {As}}_2$-${\hbox {As}}_3$ atoms forming one-dimensional zigzag chains along the *b* direction. The long ${\hbox {As}}_2$-${\hbox {As}}_3$ distance corresponds to the interchain distance and is 3.0383 Å. In ${\hbox {Ca}}_{1-x}{\hbox {La}}_x{\hbox {FeAs}}_2$, the corresponding long and short distances are 2.53 and 3.02, respectively^1^. The directional covalent bonds between the As atoms are in a zigzag form.

The complex network of atoms seen in Fig. 1b also appears in other 112-type layered iron-pnictides with the chemical formula ${\hbox {RETAs}}_2$ (T = Ag, Au)^20^. Although the space group *Pmcn* (No. 62) of ${\hbox {RETAs}}_2$ is different than those of ${\hbox {EuFeAs}}_2$ and ${\hbox {Ca}}_{1-x}{\hbox {La}}_x{\hbox {FeAs}}_2$, the compound ${\hbox {SmAuAs}}_2$ has similar structural ${\hbox {Au}}_2{\hbox {As}}_2$ and As zigzag layers^20^. In addition, the compound ${\hbox {LaAgAs}}_2$, which crystallizes in the space group *Pmca* (No.57), also consists of the ${\hbox {Ag}}_2{\hbox {As}}_2$ and As cis-trans layers^20^. These As cis–trans chain layers have only recently been discovered in iron-based pnictides^7^. It is feasible that different complex networks of As atoms, because of their 4$p^3$ electronic states, can potentially lead to discovering new iron-based pnictides and superconductors.

**Table 1:** Structural information on ${\hbox {EuFeAs}}_2$^7^.

Crystal system			
Space group	*Imm*2 (No. 44)		
*a*	21.285(9) Å		
*b*	3.9082(10) Å		
*c*	3.9752(9) Å		
$\alpha$, $\beta$, $\gamma$	$90^\circ$, $90^\circ$, $90^\circ$		

Element	*x*	*y*	*z*
Eu	0.38602 (3)	1	0.75155 (17)
${\hbox {As}}_1$	0.31613 (7)	$\frac{1}{2}$	0.2521 (6)
${\hbox {As}}_2$	$\frac{1}{2}$	$\frac{1}{2}$	0.6730 (7)
${\hbox {As}}_3$	$\frac{1}{2}$	1	0.2583 (11)
Fe	0.24970 (10)	0	0.2489 (9)

### Valence charge density maps

**Figure 2:**
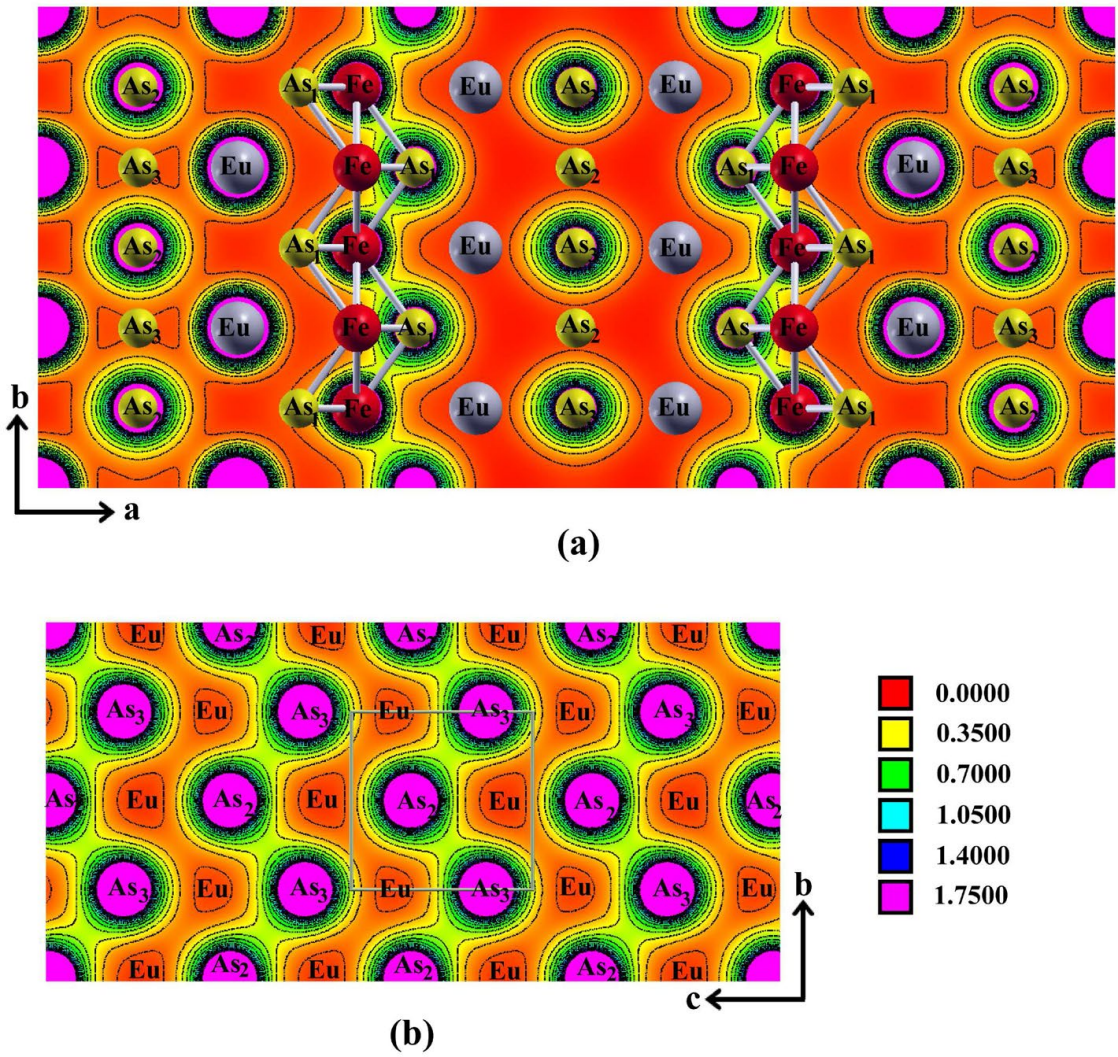
Electron charge density distribution (in units of *e*/Å$^3$) along the (001) (**a**) and (200) (**b**) planes. SigmaPlot v14, www.systatsoftware.com.

Figure 2 shows the charge density distributions in the range 0.0−1.75 *e*/Å$^3$ along two crystallographic planes in ${\hbox {EuFeAs}}_2$. The electronic charges are calculated through Bader’s analysis scheme^21^. In Fig. 2a, one observes along the elongated direction of the unit cell multiple regions in which the absence of charge density (red regions) is evident. The insulating layers are composed of the Eu atoms blocking any charge transport along the *a* direction. On the contrary, the chains of the Fe and ${\hbox {As}}_1$ atoms form covalent bonds, and thus the regions of higher valence charge density are observed (green regions). This can be related to the Fe 3*d* and As 3*p* hybridization and may indicate that the electric charge transport will, in general, be along these chains.

One also notes (Fig. 2a) that in addition to covalent (directional) bonding between the Fe and ${\hbox {As}}_1$ atoms, there are also metallic bonds between two adjacent Fe atoms. They result from the broadening of the Fe 3*d* energy levels near the Fermi level (*vide infra*). These bonds are indicated by the connecting rods in Fig. 2a. Since the Fe atoms do not lie along the (001) plane shown here, the bonds are not shown in the charge density maps. However, this does not indicate that these bonds do not exist. Instead, they are absent in the (001) plane. The charge distribution around the Eu sites and the ${\hbox {As}}_2$ sites, in the far left and far right of the unit cell, indicates that the bonding between Eu and ${\hbox {As}}_2$ is mainly ionic. In general, the valence charge density distributions indicate the coexistence of ionic bonding (specifically, due to the Eu atoms, and to a lesser extent, the ${\hbox {As}}_2$ and ${\hbox {As}}_3$ atoms) and covalent bonding (originating from the layers of the Fe and ${\hbox {As}}_1$ atoms along the *b* direction).

In Fig. 2b, the boundaries of the unit cell along the *a* direction are indicated. One finds that the charge density between the ${\hbox {As}}_2$ and ${\hbox {As}}_3$ atoms along the *b* direction is higher than that along the *c* direction. The reason for this can be traced back to the insulating role of the Eu atoms. This suggests covalent bonds between the ${\hbox {As}}_2$ and ${\hbox {As}}_3$ atoms. The valence states of both atoms are *s*-type and *p*-type, indicating the possibility of forming directional bonds. The presence of such bonds in the zigzag chains of the ${\hbox {As}}_2$ and ${\hbox {As}}_3$ atoms was also found from the analysis of the synchrotron X-ray diffraction spectra of ${\hbox {Ca}}_{1-x}{\hbox {La}}_x{\hbox {FeAs}}_2$^1^. The color contour maps in Fig. 2b clearly show the charge density distributions of regular zigzag As chains. The 4*p* orbitals between ${\hbox {As}}_2$ and ${\hbox {As}}_3$ overlap, forming the directional bonds in the zigzag chain. In contrast, there are no significant charge densities between the interchain As atoms, which indicates the absence of chemical bonds. Even stronger arguments regarding the type of chemical bonds based on the calculated density of states are given later (*vide infra*).

As mentioned above, Eu in ${\hbox {EuFeAs}}_2$ is in the divalent state^9,22^. The charge density maps indicate the electronic charge transfer from the Eu to the Fe-As layers. In other words, the ionic bonding is the consequence of this transfer, resulting in the ${\hbox {Eu}}^{2+}$ and [${\hbox {FeAs}}_2$]$^{2-}$ ions. One may also interpret this result by comparing the relatively large differences in the electronegativities of the Eu (1.2) and the As (2.18) and Fe (1.83) atoms. Likewise, the formation of covalent bonds between the As and Fe atoms results from their similar electronegativity values.

With the above valence charge transfer analysis, one may express the chemical formula ${\hbox {EuFeAs}}_2$ as ${\hbox {Eu}}^{2+}$(${\hbox {Fe}}_2^{2+}{\hbox {As}}_2^{3-}$)$_{1/2}{\hbox {As}}^-$. The first of the two Eu 6*s* electrons is transferred to the Fe–${\hbox {As}}_1$ layer and the other to the As zigzag layer. Two Fe 4*s* electrons, together with a single Eu 6*s* electron, are transferred to the Fe–${\hbox {As}}_1$ layer. Their related orbitals overlap with three empty ${\hbox {As}}_1$ 4*p* orbitals, shaping covalent bonds in the Fe–${\hbox {As}}_1$ layers.

The As zigzag bond layers are composed of ${\hbox {As}}^-$ ions with the 4$p^4$ electronic states. Because the crystal field around the As ions is anisotropic, the threefold degeneracy of the ${\hbox {As}}^-$ 4*p* orbitals is expected to be removed. The ${\hbox {As}}_2$ and ${\hbox {As}}_3$ ions are closely packed along the *bc* plane. This causes the $p_x$ orbital with two electrons to become stabilized due to the large spatial overlap. In contrast, the $p_y$ and $p_z$ orbitals with one unpaired electron become destabilized due to their small overlap. The latter two orbitals spread along the *bc* plane, forming the 45$^\circ$ angles with the *b* and *c* directions. Thus, one predicts that the zigzag pattern is formed by directional bonding between the ${\hbox {As}}_2$ and ${\hbox {As}}_3$ atoms in close proximity.

### Density of states

Using density functional theory, we investigate the electronic, magnetic, and hyperfine-interaction properties of the 112-type iron-pnictide compound ${\hbox {EuFeAs}}_2$.

**Figure 3:**
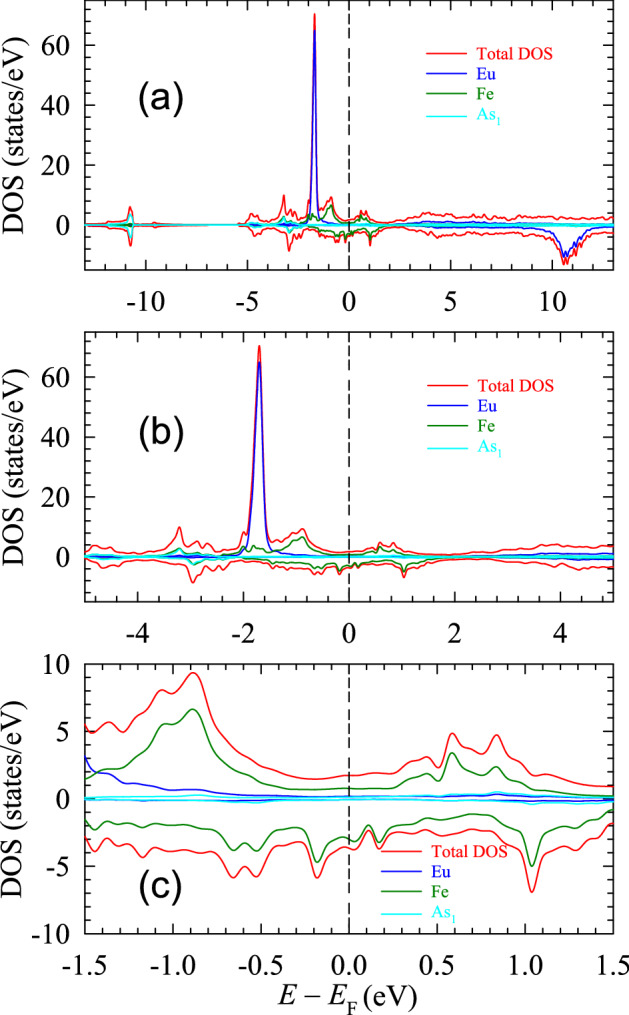
Spin-resolved, total and atom-resolved DOS in ${\hbox {EuFeAs}}_2$ for the energy range between $-13$ and 13 eV (**a**), $-5$ and 5 eV (**b**), and $-1.4$ and 1.4 eV (**c**). SigmaPlot v14, www.systatsoftware.com.

We performed density of states (DOS) calculations using the modified tetrahedron method^23^. Figure 3 shows the spin-resolved, total and atom-resolved (except for the ${\hbox {As}}_2$ and ${\hbox {As}}_3$ atoms) DOS of ${\hbox {EuFeAs}}_2$. The DOS calculated over the wide energy range is displayed in Fig. 3a. One observes a sharp peak at $-1.7$ eV, which originates from Eu’s majority spin states. These Eu 4*f* states are highly localized as their energy width is small. The Eu’s minority spin states, on the other hand, peak at around 10.5 eV. Although they are broader than the corresponding majority spin states, they are nonetheless confined to an energy interval 9.5–11.7 eV. The energy difference between these two spin states results from the large value of the effective Hubbard parameter (7.48 eV) used for Eu, which shifts the minority spin states out of the Fermi energy region. Since these minority spin states (spin-down states) lie high in energy, no electron can potentially be excited into them. Consequently, they remain empty and play no role in the physical properties of the compound studied.

By comparing the Eu’s majority and minority spin state DOS (Fig. 3a), one finds a large difference between the maximum DOS for these two components (Table 2. This difference leads to a relatively large magnetic moment of $6.9~\mu _{{\text {B}}}$ carried by the Eu atoms.

**Table 2:** The total and partial DOS (in states/eV) in ${\hbox {EuFeAs}}_2$.

Number of states at the Fermi level	Spin up	Spin down
*N*(${\hbox {EuFeAs}}_2$)	1.81626	3.74682
*N*(Eu)	0.22862	0.00282
*N*(Fe)	0.80444	2.98899
*N*(Fe 3*d*)	0.34999	1.54276
*N*(Fe $d_{z^2}$)	0.07564	0.37144
*N*(Fe $d_{x^2-y^2}$)	0.07564	0.15241
*N*(Fe $d_{xy}$)	0.07564	0.37144
*N*(Fe $d_{xz}$)	0.01693	0.15241
*N*(Fe $d_{yz}$)	0.10500	0.37144
*N*(${\hbox {As}}_1$)	0.14352	0.06732
*N*(${\hbox {As}}_2$)	0.04445	0.05461
*N*(${\hbox {As}}_3$)	0.04445	0.05461

The calculated DOS allows one to deduce qualitatively the nature of chemical bonding in which the Eu atoms participate. Because the overlap region (Fig. 3a) of the Eu DOS with the DOS of Fe and As is small (due to its highly localized DOS profile), one can speculate that the Eu atoms participate in forming ionic bonds with the Fe and As atoms and to a lesser extent weaker covalent bonds. This agrees with the earlier conclusion that was based on the electron charge density distributions. Also, one can see that the Eu DOS is negligible at $E_{{\text {F}}}$ and in the $E_{{\text {F}}}$ region. Therefore, one expects part of the insulating properties of ${\hbox {EuFeAs}}_2$ to emerge from the lack of Eu *f* states in the Fermi region.

The Fe DOS spans the region from $-3.5$ to 1.8 eV (Fig. 3b). It originates mainly from the Fe 3*d* states, which are relatively delocalized in energy and dominate the $E_{{\text {F}}}$ region. Therefore, the electrical properties of ${\hbox {EuFeAs}}_2$ can be related to these states. The Fe spin-up states peak at $-3.2$, $-1.7$, $-0.9$, and 0.6 eV, whereas the spin-down states peak at $-0.6$, $-0.2$, and 1.0 eV. The DOS profiles of the Fe spin-up and spin-down states are asymmetric both in the number of states (Table 2) and in the location in the energy spectrum, which explains the nonzero Fe magnetic moment ($0.78~\mu _{\text B}$). A careful inspection of Fig. 3b shows a relatively large energy gap in the spin-up states and a pseudogap in the spin-down states across the $E_{{\text {F}}}$ region.

A closeup plot of the DOS in the $E_{{\text {F}}}$ region is shown in Fig. 3c. One can notice that the DOS in the vicinity of $E_{{\text {F}}}$, although very small, originates mainly from the Fe states. The spin-down Fe states show a minimum slightly above $E_{{\text {F}}}$ (at 0.1 eV), similar to what is observed in other Fe–As containing compounds^24,25^.

**Figure 4:**
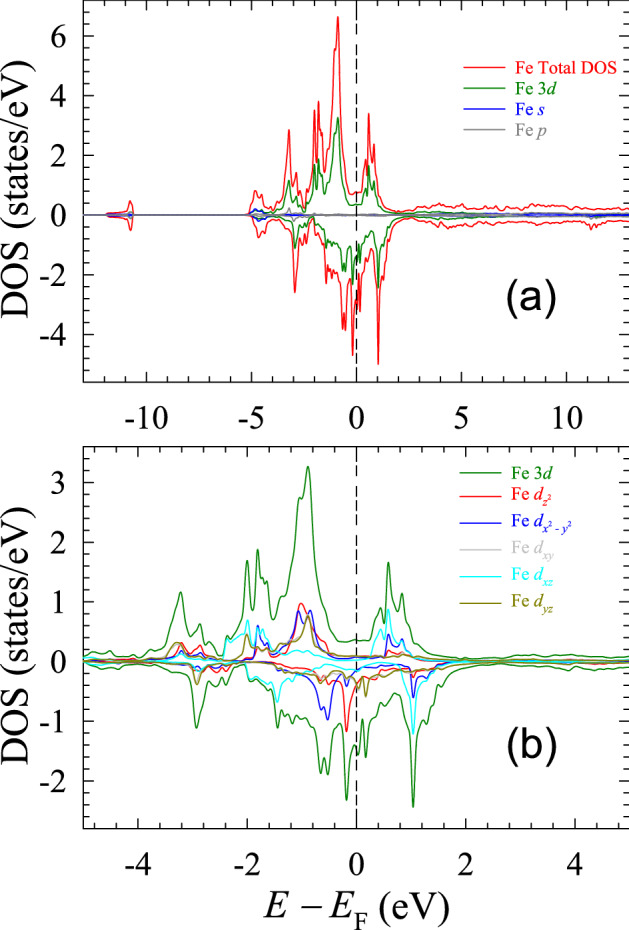
Spin-resolved, Fe total and orbital-resolved DOS (**a**) and Fe *d*-orbital-resolved DOS (**b**). SigmaPlot v14, www.systatsoftware.com.

Figure 4a displays the spin-polarized, total and orbital-resolved DOS of Fe in a wide energy range between $-13$ and 13 eV. One notices that the Fe 3*d* states dominate the DOS in the $E_{{\text {F}}}$ region. In Fig. 4b, we examine the spin-polarized DOS of the five orbitals of the Fe 3*d* states. The maximum peak in the spin-up DOS at $-0.9$ eV arises mainly from the $d_{z^2}$, $d_{x^2-y^2}$, $d_{yz}$, and $d_{xy}$ states. The $d_{xz}$ orbital contributes to the DOS mainly above $E_{{\text {F}}}$. The states originating from this orbital lead to the peaks in the spin-up DOS at $-2.0$, 0.6, and 1.0 eV. The $d_{z^2}$ states, together with the $d_{xz}$ states, give rise to the DOS in the vicinity of $E_{\text {F}}$. Most of the electronic properties of ${\hbox {EuFeAs}}_2$ result from them. Close to the semi-core energy region (between $-3.2$ and $-2.9$ eV), one finds peaks arising from the $d_{xy}$ and $d_{yz}$ orbitals.

**Figure 5:**
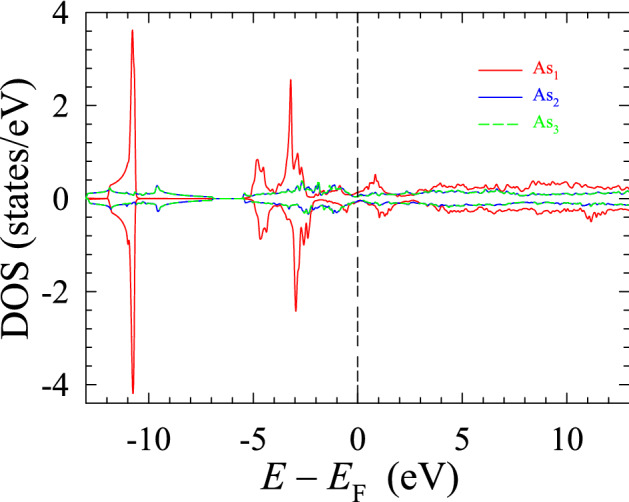
Spin-resolved DOS of As. SigmaPlot v14, www.systatsoftware.com.

The spin-polarized DOS of the As atoms located at three crystallographic positions (Table 1) is shown in Fig. 5. It is evident that the dominant contribution to the DOS comes from the ${\hbox {As}}_1$ atoms. The deep core states, which are the atomic-like states, peak at $-10.7$ eV. They do not contribute to the physical and chemical properties of the compound studied. One observes two peaks (Fig. 5): one at $-3.2$ eV from the spin-up states, and the other at $-2.9$ eV from the spin-down states. These peaks coincide with the Fe peaks deriving from the $d_{xy}$ and $d_{yz}$ orbitals (Fig. 4b). There are two smaller peaks in the vicinity of $E_{\text {F}}$ (Fig. 5) at $-0.9$ and 0.8 eV. They also overlap with the peaks of the Fe *d* orbitals. These overlaps can explain the chemical bonding between the Fe and ${\hbox {As}}_1$ atoms, thus confirming the earlier conclusion based upon the electron charge density maps and the structure of the ${\hbox {EuFeAs}}_2$ compound. Moreover, it can also be seen (Fig. 5) that the DOS stemming from the ${\hbox {As}}_2$ and ${\hbox {As}}_3$ atoms completely overlaps with each other throughout a wide energy range, and its contribution to the total As DOS is minute. This significant amount of overlap over a wide energy range is the signature of covalent bonding between the two As atoms because such states are highly delocalized in energy. This observation confirms an earlier conclusion based on the analysis of the charge density maps (Fig. 2b).

As mentioned earlier, the ${\hbox {As}}_2$ and ${\hbox {As}}_3$ atoms do not form bonds with the Fe atoms. The absence of such bonds can be partially due to a large separation between these As and Fe atoms. Therefore the hybridization between these As atoms and the Fe–${\hbox {As}}_1$ layers is negligible, as observed from the DOS plots.

Another important feature of Fig. 5 is the near-perfect symmetry of the spin-up and spin-down DOS profiles. This accounts for the near-zero magnetic moment of the As atoms in the compound studied. For regions above $E_{\text {F}}$ (typically above 2.0 eV), the As atoms’ DOS profile is similar to that of a quasi-free electron gas. This similarity is mainly because these states are related to conduction bands, and because of their high energy, they are unoccupied. In Table 1, the values of the contributions to DOS of various atoms and orbitals are given.

### Energy band structure

**Figure 6:**
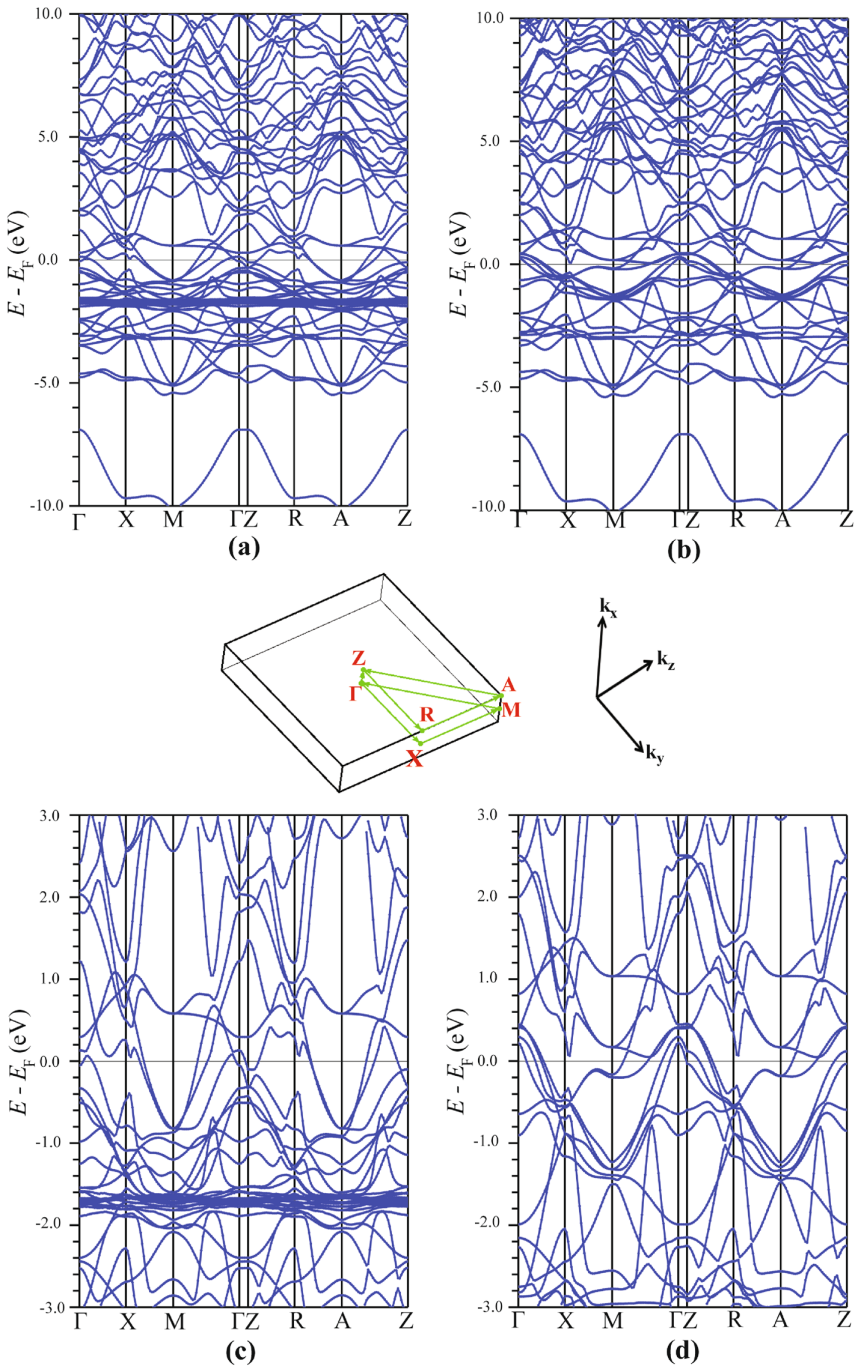
Spin-polarized band structure of ${\hbox {EuFeAs}}_2$ in the high-symmetry directions of the conventional Brillouin zone. The spin-up (**a**) and spin-down (**b**) band structure in the energy range between $-10$ and 10 eV. The spin-up (**c**) and spin-down (**d**) band structure close to the Fermi level. The **k**-paths along the high symmetry directions are shown in the middle of the figure. SigmaPlot v14, www.systatsoftware.com.

The spin-resolved energy band structure of ${\hbox {EuFeAs}}_2$ is presented in Fig. 6. It is plotted along the **k**-paths passing through high symmetry directions in the conventional Brillouin zone shown in the middle of the figure. One notices in Fig. 6a that there is a dense concentration of nearly flat bands around 1.7 eV. These bands are related to the strongly localized Eu 4*f* states. As one moves in the Brillouin zone from one symmetry point to another, they show almost zero dispersion. As the effective mass $m^*$ of the carriers is inversely proportional to the band curvature, $m^* \sim [\nabla _\mathbf{k }^2E(\mathbf{k })]^{-1}$, the electrons in these localized states cannot participate in transport properties, and are essentially immobile. The numerous packed bands arising from the Eu 4*f* states verify the presence of a very high DOS peak in Fig. 3a.

Another feature of the band structure (Fig. 6) is the presence of a highly dispersive region extending from $\sim 2$ eV to higher energies. All bands in this region are the practically empty conduction bands. Their large dispersion indicates that they are of the *s* and *p* character and mostly derive from the Eu and As atoms. Below $E_{\text {F}}$ (Fig. 6a), and except for the flat Eu bands, one finds a region of less dispersive bands. These bands are primarily related to the Fe and ${\hbox {As}}_1$ states. Between $-5$ and $-4$ eV (Fig. 6a), a couple of energy bands are observed, which arise from the As semi-core states. Below $-5$ eV, a large gap separates the semi-core bands from a single-core band lying deep in energy.

Figure 6b is the spin-down counterpart of Fig. 6a. Its features are similar to those of Fig. 6a discussed above. There are, however, some differences. First, the spin-down Eu 4*f* states are pushed, due to the large Hubbard parameter, far above the $E_{\text {F}}$ region to energies higher than 10 eV. Second, several energy bands are crossing $E_{\text {F}}$. They originate from the Fe 3*d* states, in agreement with the results from the DOS calculations (Fig. 3a).

As the energy band structure, and the DOS, in the proximity of $E_{\text {F}}$ determine most of the physical and chemical properties of the compound, we display the spin-up and spin-down energy band structure of ${\hbox {EuFeAs}}_2$ in the energy range between $-3$ and 3 eV in Fig. 6c,d, respectively. These band structures show some features also observed in other Fe-based pnictides^26–30^. However, there are some differences. There are four hole-like bands at $\Gamma$(0,0) (Fig. 6d): one peak at 0.2 eV, the next one at 0.3 eV, and the two other bands become degenerate and peak at 0.4 eV. This is different from what was found for such a pnictide as ${\hbox {BaFe}}_2{\hbox {As}}_2$, where three hole-like bands appear near the $\Gamma$ point^31^. Two electron-like bands at point M($\pi$,$\pi$) are seen in Fig. 6c, which is similar to what is observed in another Fe-based compound LaFeAsO^31^. This figure also shows that the two electron-like bands merge at points M and A, where the Brillouin zone is highly symmetric, and thus the states become degenerate at these points. As one moves away from these high-symmetry points, the bands start to diverge from each other. The hole-like band around the highest symmetry point $\Gamma$ is also seen to cross the $E_{\text {F}}$.

One notices in Fig. 6d that the number of bands crossing the $E_{\text {F}}$ is larger than for the spin-up case (Fig. 6c). Moreover, in both figures, the highly dispersive conduction bands above 1 eV can be clearly seen, in contrast to the less dispersive Fe 3*d* bands dominating the $E_{\text {F}}$ region (between $- 1$ and 1 eV).

**Figure 7:**
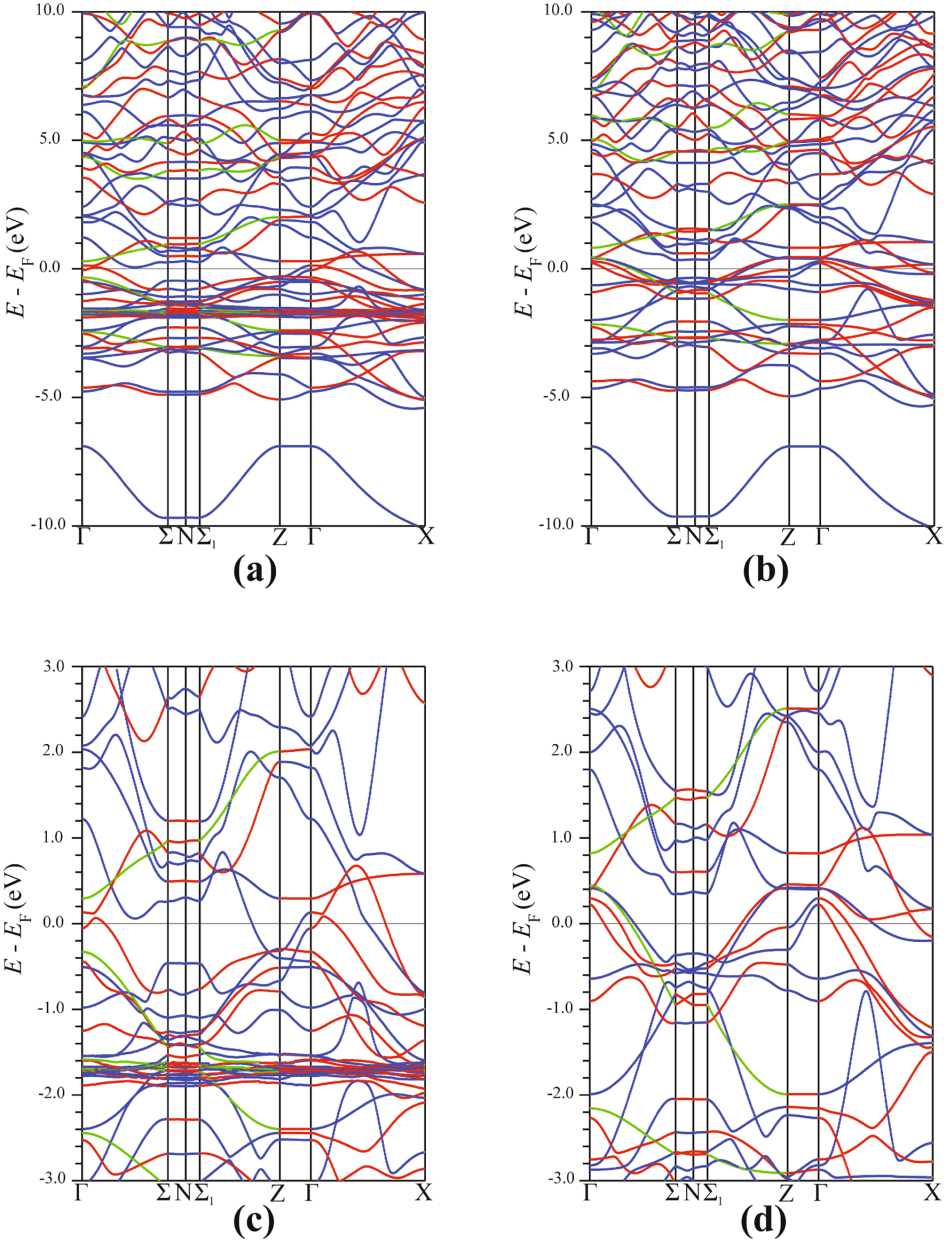
Spin-polarized band structure of ${\hbox {EuFeAs}}_2$ in the high-symmetry directions of the conventional Brillouin zone. The spin-up (**a**) and spin-down (**b**) band structure in the energy range between $-10$ and 10 eV. The spin-up (**c**) and spin-down (**d**) band structure close to the Fermi level. SigmaPlot v14, www.systatsoftware.com.

Similar to other Fe-based pnictides, the electronic structure of ${\hbox {EuFeAs}}_2$ is mainly determined by the Fe-As layers. There is, however, a distinctive difference between the band structure of ${\hbox {EuFeAs}}_2$ and that of other known Fe pnictides. Figure 7 depicts the spin-resolved energy band structure along the **k**-path $\Gamma -\Sigma - {\text {N}} - \Sigma _1 - \text {Z} - \Gamma -\text {X}$ in the first Brillouin zone. There are very few dispersive bands crossing $E_{\text {F}}$. Qualitatively, the same feature observed in Fig. 6 can also be seen in the band structure along this other **k**-path. An important observation here is that the hole-like bands are concentrated along the $\Gamma$ direction. In contrast, the electron-like bands are found around the N and X directions. Since a few bands crossing the Fermi level (Fig. 7), one may conclude that insulating properties of ${\hbox {EuFeAs}}_2$ are more dominant than the conducting ones. Thus, one can categorize ${\hbox {EuFeAs}}_2$ as an insulator or a weak conductor.

**Figure 8:**
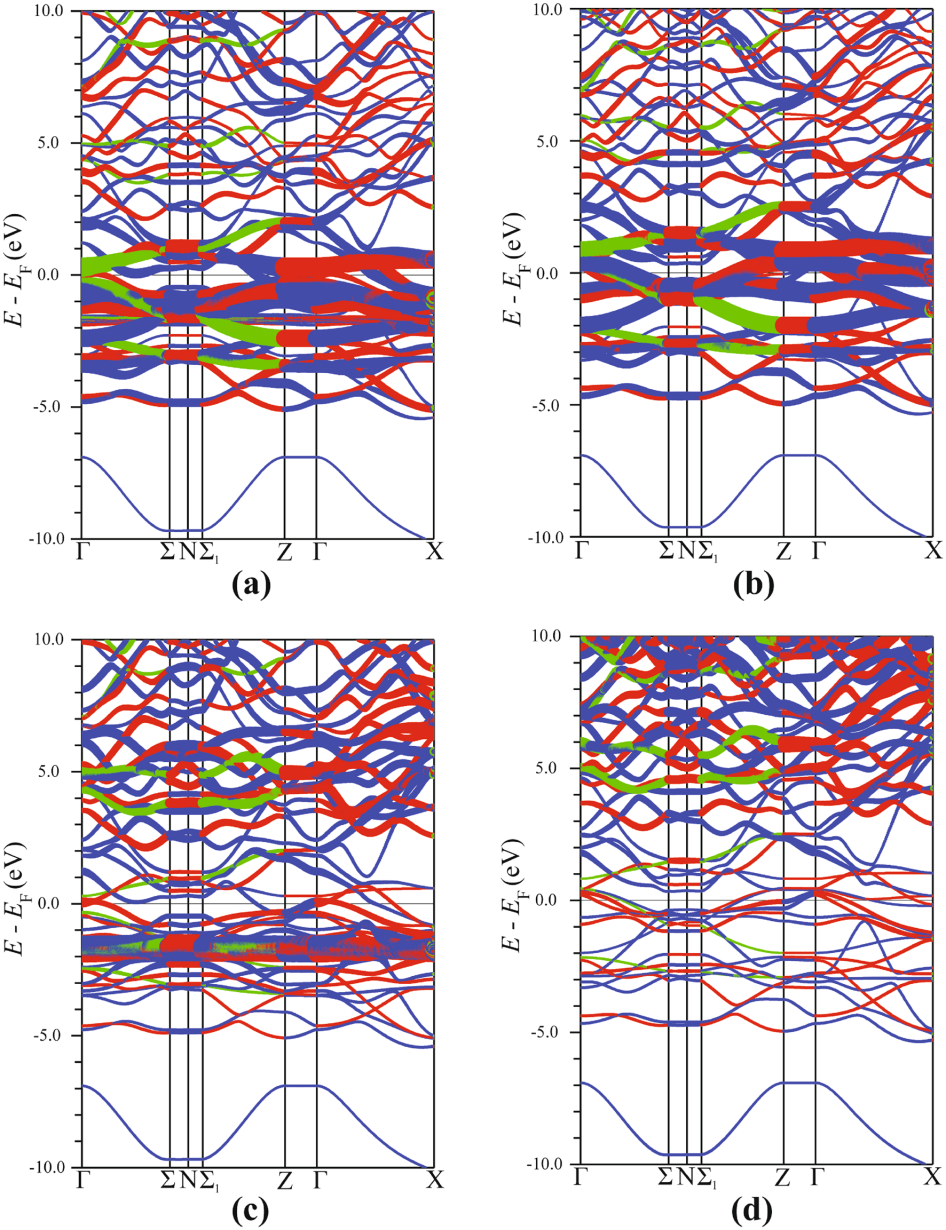
Spin-polarized, weighted energy band structure of ${\hbox {EuFeAs}}_2$. The spin-up (**a**) and spin-down (**b**) band structure of Fe. The spin-up (**a**) and spin-down (**b**) band structure of Eu. SigmaPlot v14, www.systatsoftware.com.

Figure 8 shows the weighted Fe and Eu energy bands. The thickness of the bands represents their ”weight” in the Brillouin zone. It is clear that the $E_{\text {F}}$ region is dominated by the Fe bands originating from the 3*d* states and that Eu 4*f* bands do not appear in this region. Therefore, one can state that the Fe 3*d* bands essentially determine the physical and chemical properties of ${\hbox {EuFeAs}}_2$.

**Figure 9:**
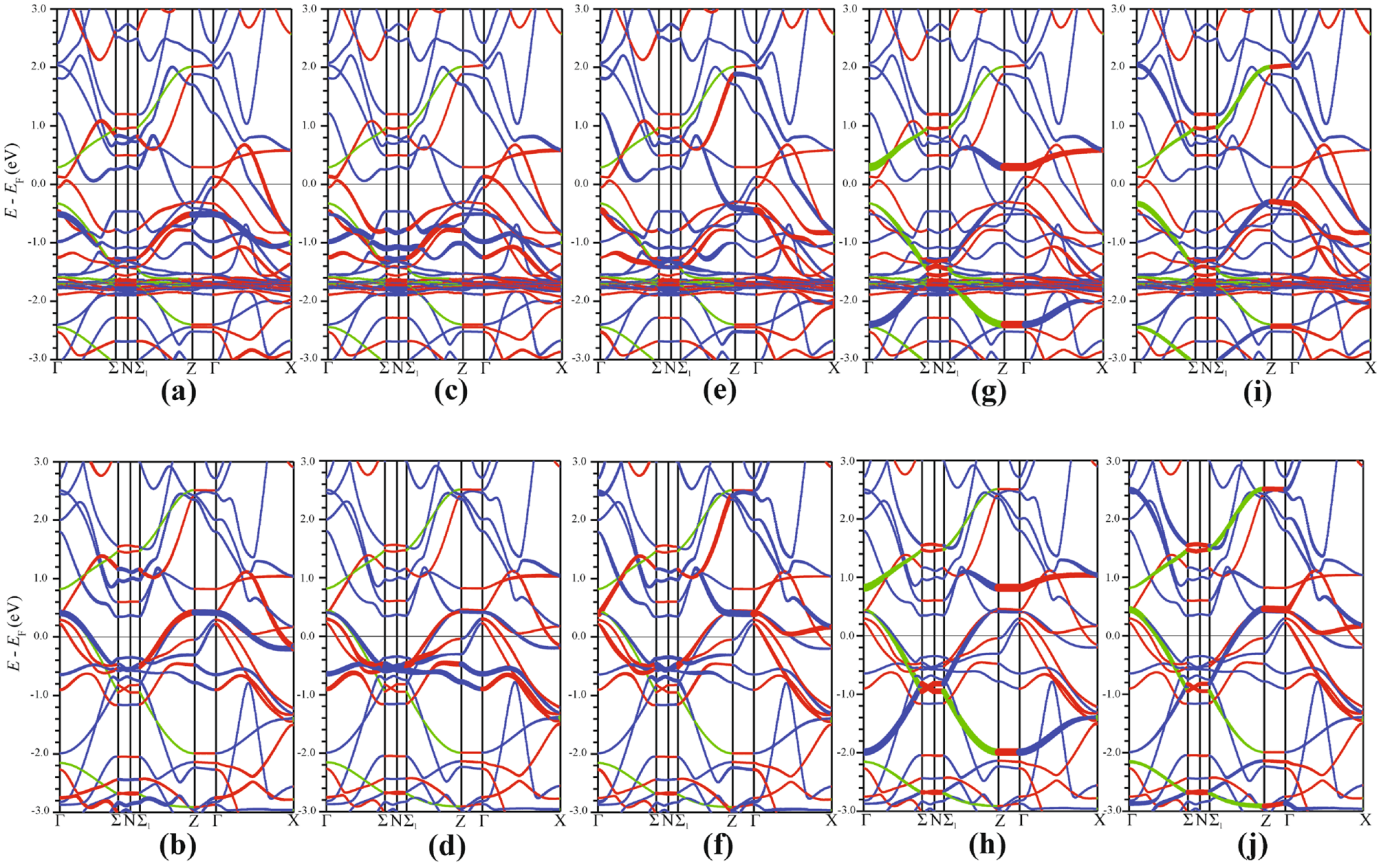
Spin-polarized, Fe 3*d* orbital-resolved band structure shown by weighted bands in the background of the ${\hbox {EuFeAs}}_2$ energy bands. The $d_{z^2}$ contribution to the spin-up (**a**) and spin-down (**b**) energy bands. The $d_{x^2-y^2}$ contribution to the spin-up (**c**) and spin-down (**d**) energy bands. The $d_{xy}$ contribution to the spin-up (**e**) and spin-down (**f**) energy bands. The $d_{xz}$ contribution to the spin-up (**g**) and spin-down (**h**) energy bands. The $d_{yz}$ contribution to the spin-up (**i**) and spin-down (**j**) energy bands. SigmaPlot v14, www.systatsoftware.com.

The spin-resolved energy band structure of the five Fe 3*d* orbital-resolved states is shown in Fig. 9. All Fe bands are depicted as ”thick” bands in other bands’ background (the band thickness indicates its relative weight with respect to the overall band structure of ${\hbox {EuFeAs}}_2$). The spin-up portion of the $d_{z^2}$ band (Fig. 9a) lies below $E_{\text {F}}$, whereas the spin-down portion crosses $E_{\text {F}}$ at mid-point along the $\Gamma - \text {X}$, $\text {Z} - \Sigma _1$, and $\Gamma - \Sigma _1$ directions. The contribution from the $d_{z^2}$ orbital to the band crossing of $E_{\text {F}}$ is almost negligible (Fig. 9c,d). The bands associated with the $d_{xz}$ orbitals (Fig. 9g,h) are somewhat away from $E_{\text {F}}$. The bands originating from the $d_{yz}$ orbital (Fig. 9i,j) are degenerate with those emerging from the $d_{z^2}$ orbital at the $\Gamma$ and $\text {Z}$ points.

### Fermi surface topology

Among the numerous energy bands in ${\hbox {EuFeAs}}_2$, only a few contribute to the Fermi surface, and the majority of the energy bands show negligible dispersion at the Fermi level.

As has been observed in the previous sections, the electronic properties of ${\hbox {EuFeAs}}_2$ depend predominantly on the electronic structure in the $E_{\text {F}}$ region. In particular, the energy bands and occupied states in the proximity of $E_{\text {F}}$ determine most of the compound’s electronic transport properties. This highlights the importance of the fermiology of the compound. The Fermi surfaces (or Fermi sheets) are branes in the reciprocal space that separate the unoccupied states from the occupied ones and constitute constant energy(the Fermi energy) regions. Most properties of a compound stem from the size and shape of these surfaces.

**Figure 10:**
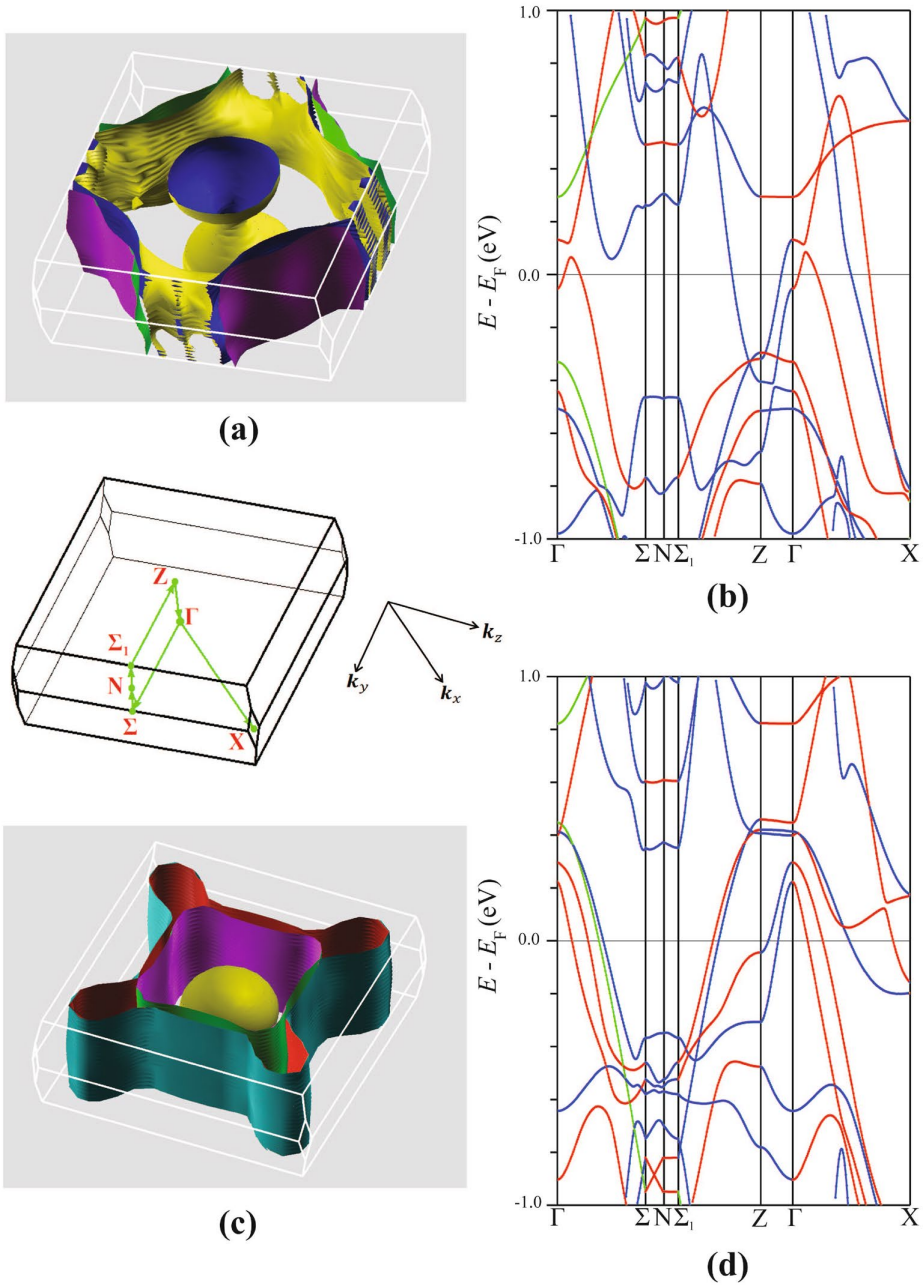
Spin-up (**a**) and spin-down (**c**) Fermi surfaces of ${\hbox {EuFeAs}}_2$. The corresponding near-Fermi energy band structure for spin-up (**b**) and spin-down (**d**) energy bands. The corresponding **k**-path traversed in the primitive Brillouin zone is shown at the center of the figure. SigmaPlot v14, www.systatsoftware.com.

Figure 10 displays the spin-resolved Fermi surface sheets in the primitive Brillouin zone together with the corresponding band structures in the neighbourhood of $E_{\text {F}}$. Qualitatively, the resulting surface topology agrees with the band structure calculations. For the spin-up case, one observes the presence of a hole-like band in the center of the Brillouin zone along the $\text {Z} - \Gamma$ path (Fig. 10b) corresponding to the Fermi sheet in the central region (Fig. 10a). Moreover, as one moves along the $\Gamma - \text {X}$ direction, a hole-like and two electron-like bands cross $E_{\text {F}}$ (Fig. 10b) and the emerging Fermi sheets are shown at the corners of the Brillouin zone (Fig. 10a). One observes two electron pockets along the four corners of the Brillouin zone. Along the $\Gamma - \Sigma - \text {N}$ path, the hole-like sheets (Fig. 10a) corresponding to the hole-like bands (Fig. 10b) can also be seen.

Figure 10c,d show the spin-down Fermi surface topology and the energy bands, respectively. The hole-like sheets along the $\Gamma - \text {X}$ and $\text {Z} - \Gamma$ directions can be observed in Fig. 10c. These sheets correspond to the hole-like bands in Fig. 10d. A close inspection of Fig. 10c,d shows that starting from the $\Gamma$ point and moving towards the $\Sigma$ point, the first two hole surfaces related to the two hole-like bands (which peak at 0.2 and 0.3 eV) are in a shape of an oval and a square cylinder, respectively. The two nearly degenerate hole-like bands located midway along the $\Gamma - \Sigma$ path correspond to the two barely resolved adjacent hole-like sheets (Fig. 10c). By careful comparison of these Fermi surfaces with the Fe orbital-resolved bands in Fig. 9, one concludes that by traversing the Brillouin zone from $\Gamma$ to $\Sigma$, the four sheets described above derive from the Fe $d_{xy}$, $d_{x^2-y^2}$, $d_{yz}$, and $d_{z^2}$ orbitals, respectively.

### Magnetic moments and Mössbauer hyperfine parameters

Each formula unit of ${\hbox {EuFeAs}}_2$ contains two Eu atoms, two ${\hbox {As}}_1$ atoms, one ${\hbox {As}}_2$ atom, one ${\hbox {As}}_3$ atom and two Fe atoms. The calculated magnetic moments are listed in Table 3. The total magnetic moment per formula unit (15.43822 $\mu _ {\text {B}}$) consists of the contribution from the muffin-tin region (15.16166 $\mu _ {\text {B}}$) and that from the interstitial region (0.27656 $\mu _ {\text {B}}$). The calculated magnetic moment of Fe (0.78029 $\mu _ {\text {B}}$) is in excellent agreement with the experimental value of 0.78(1) $\mu _ {\text {B}}$ estimated from the Mössbauer data^9^. It is evident (Table 3) that the dominant contribution to the magnetic moment of ${\hbox {EuFeAs}}_2$ derives from the Eu atoms.

**Table 3:** Calculated magnetic moments (in $\mu _ {\text {B}}$).

Element	Magnetic moment
Eu	6.90059
${\hbox {As}}_1$	− 0.07118
${\hbox {As}}_2$	− 0.02881
${\hbox {As}}_3$	− 0.02892
Fe	0.78029
Muffin-tin region	15.16166
Interstitial region	0.27656
Total	15.43822

We note here that the studied compound crystallizes in the *Imm*2 space group, whereas the majority of iron-based pnictides crystallize in the $P2_1$/*m* space group. The *Imm*2 space group can result in relatively localized magnetic moments, which are usually robust. On the other hand, the $P2_1$/*m* space group can lead to very itinerant magnetism in iron-based pnictides, making the calculated magnetic energies extremely sensitive to the type of approximations used in the DFT calculations. It is also known that GGA calculations increase the tendency towards magnetism as they underestimate spin fluctuations. We have not optimized the lattice and atomic position parameters for the current compound but used their experimental values. There are studies that relate the bonding angle between the As and Fe atoms in the Fe layers to the Fe magnetic moment^32^. It seems that the 108.212$^\circ$ bonding angle may be a factor leading to a relatively small Fe magnetic moment in ${\hbox {EuFeAs}}_2$.

Calculations carried out for antiferromagnetic configurations, where the Eu magnetic moments are confined to the *b*–*c* plane and change direction along the alternating layers along the *a* direction, show an increase in the Fe magnetic moment. This magnetic moment is $\sim 1.8$ and $\sim 2.1 \mu _ {\text {B}}$ for the antiferromagnetic checkboard and stripe configurations, respectively.

To understand the magnetic interactions in the Eu layers of the compound studied, we apply an effective Heisenberg model, ${\hat{H}} = J_{1} \sum _{(i,j)^{}} \mathbf{S} _{i} \cdot \mathbf{S} _{j} + J_{2} \sum _{(i,j)^{}} \mathbf{S} _{i} \cdot \mathbf{S} _{j}$. Here $\mathbf{S} _{i}$($\mathbf{S} _{j}$) is the spin of the Eu atom identified by *i*(*j*) and the summation is over nearest neighbors. The first term with the coupling constant $J_1$ indicates interactions between the in-plane Eu atoms, while the second term with the coupling constant $J_2$ describes interactions between the Eu atoms along the *a* direction. The calculated values for $J_1$ and $J_2$ are 89 $\mu$Ry and 6.6 $\mu$Ry, respectively.Also, the calculated values for the Fe–Eu nearest-neighbor interactions give a coupling constant of 82 $\mu$Ry for the *d*–*f* exchange interaction. Using the $108.212^\circ$ bonding angle between the As and Fe atoms in the Fe layers, we calculated the Fe layers’ spin exchange to the nearest-neighbor and next-nearest-neightbor to be 2.2 mRy and 0.7 mRy, respectively. The calculated magnetic exchange interaction between Fe nearest-neighbor atoms is 65 mRy.

We also calculated three hyperfine-interaction parameters, which can be derived from the Mössbauer spectra. These parameters are the isomer shift $\delta _0$, the hyperfine magnetic field $H_ \text {hf}$, and the principal component of the electric field gradient tensor $V_{zz}$^33,34^.

The isomer shift is calculated from the following relation: $\delta _0 = \alpha [\rho (0) - \rho _{\text {ref}}(0)]$. Here, $\rho (0)$ and $\rho _{\text {ref}}(0)$ are, respectively, the total electron densities at the Mössbauer nucleus in the compound studied and in the reference material, and $\alpha$ is a calibration constant. To account for the possibility of the penetration of the $p_{1/2}$ electrons into the $^{57}$Fe nuclei, relativistic spin-orbit effects were included in the calculations of $\rho$^35^. As a reference material for $^{57}$Fe Mössbauer spectroscopy, $\alpha$-Fe metal (with the *bcc* structure and the lattice constant of 2.8665 Å) was used. The calculated values of $\rho (0)$ and $\rho _{\text {ref}}(0)$ were found to be 15308.415 and 15309.918 $\text {(a.u.)}^{-3}$, respectively. With the calibration constant $\alpha = -0.291 \text {(a.u.)}^3$(mm/s)^36^, these $\rho$ values lead to $\delta _0 = 0.4374$ mm/s. The calculated $\delta _0$ is in fair agreement with the experimental value of 0.547(2) mm/s^9^.

There are three major contributions^33,34^to the measured $H_ \text {hf}$: the Fermi contact term $H_ {\text c}$, the magnetic dipolar term $H_ \text {dip}$, and the orbital moment term $H_ \text {orb}$. Usually, the magnitude of the first term is much greater than that of the other two terms. The first term can be calculated from the following expression: $H_ {\text c} = \frac{8\pi }{3}\mu _{\text {B}}^2(\rho _{\uparrow }(0) - \rho _{\downarrow }(0))$. Here, $\rho _{\uparrow }(0)$ and $\rho _{\downarrow }(0)$ are the spin-up and spin-down densities at the Mössbauer nucleus, respectively. The calculated magnitudes of $H_ {\text c}$ at the $^{57}$Fe and $^{151}$Eu nuclei are, respectively, 59.8 and 308.7 kOe. These compare well with the corresponding experimental values (at 0 K) of 47.6(9) and 294.2(7) kOe^9^.

And finally, the calculated $V_{zz}$ at the $^{151}$Eu nuclei is $-5.54 \times 10^{21}$ V/${\hbox {m}}^2$. It is in good agreement with the experimental value of $-4.98 \times 10^{21}$ V/${\hbox {m}}^2$^9^.

## Conclusions

We performed a comprehensive first-principles study, using density-functional theory, of a new iron-pnictide compound ${\hbox {EuFeAs}}_2$. The geometry of the crystal structure and the atomic species in ${\hbox {EuFeAs}}_2$ lead to an interconnected network of Fe and As layers. These layers are separated by sheets of As atoms and insulating layers of Eu atoms. The calculated valence charge density maps show the existence of a mixture of ionic bonds between the ${\hbox {Eu}}^{2+}$ and [${\hbox {FeAs}}_2$]$^{2-}$ ions, covalent bonds between the Fe and ${\hbox {As}}_1$ atoms, and directional bonds within the zigzag chains between the ${\hbox {As}}_2$ by ${\hbox {As}}_3$ atoms. The role of the Fe 3*d* orbitals in forming the band structure is discussed in detail. The band-structure similarities between ${\hbox {EuFeAs}}_2$ and other iron-based pnictides, as well as the unique band-structure features of ${\hbox {EuFeAs}}_2$, are elucidated. The 2D magnetism of ${\hbox {EuFeAs}}_2$ is shown to result from the spatial distribution of atoms. The magnetic moments of ${\hbox {EuFeAs}}_2$ and each constituent atom are calculated. We show that the calculated Mössbauer hyperfine-interaction parameters are in good agreement with the experimental ones.
